# Chronic NF-κB blockade improves renal angiotensin II type 1 receptor functions and reduces blood pressure in Zucker diabetic rats

**DOI:** 10.1186/s12933-015-0239-7

**Published:** 2015-06-10

**Authors:** Hao Luo, Xinquan Wang, Jialiang Wang, Caiyu Chen, Na Wang, Zaicheng Xu, Shuo Chen, Chunyu Zeng

**Affiliations:** The Department of Cardiology, Daping Hospital, The Third Military Medical University, Chongqing, PR China; Chongqing Institute of Cardiology, Chongqing, PR China

**Keywords:** Angiotensin II type 1 receptor, Inflammatory, Oxidative stress, Nuclear factor-kappa B, Hypertension

## Abstract

**Background:**

Both angiotensin II type 1 receptor (AT_1_R) and nuclear factor-kappa B (NF-κB) play significant roles in the pathogenesis of hypertension and type 2 diabetes. However, the role of NF-κB in perpetuating renal AT_1_ receptors dysfunction remains unclear. The aim of the present study to determine whether blockade of NF-κB, could reverse the exaggerated renal AT_1_R function, reduce inflammatory state and oxidative stress, lower blood pressure in Zucker diabetic fatty (ZDF) rats.

**Methods:**

Pyrrolidine dithiocarbamate (PDTC), a NF-κB inhibitor (150 mg/kg in drinking water)or vehicle was administered orally to 12-weeks-old ZDF rats and their respective control lean Zucker (LZ) rats for 4 weeks. Blood pressure was measured weekly by tail-cuff method. AT_1_R functions were determined by measuring diuretic and natriuretic responses to AT_1_R antagonist (candesartan; 10 μg/kg/min iv). The mRNA and protein levels of NF-κB, oxidative stress maker and AT_1_R were determined using quantitative real-time PCR and Western blotting, respectively. The NF-κB-DNA binding activity in renal cortex was measured by Electrophoretic mobility shift assay (EMSA).

**Results:**

As compared with LZ rats, ZDF rats had higher blood pressure, impaired natriuresis and diuresis, accompanied with higher levels of oxidative stress and inflammation. Furthermore, AT_1_R expression was higher in renal cortex from ZDF rats; candesartan induced natriresis and diuresis, which was augmented in ZDF rats. Treatment with PDTC lowered blood pressure and improved diuretic and natriuretic effects in ZDF rats; meanwhile, the increased oxidative stress and inflammation were reduced; the increased AT_1_R expression and augmented candesartan-mediated natriuresis and diuresis were recoverd in ZDF rats. Our further study investigated the mechanisms of PDTC on AT_1_R receptor expression. It resulted that PDTC inhibited NF-κB translocation from cytosol to nucleus, inhibited binding of NF-κB with AT_1_R promoter, therefore, reduced AT_1_R expression and function.

**Conclusions:**

Our present study indicates blockade of NF-κB, via inhibition of binding of NF-κB with AT_1_R promoter, reduces renal AT_1_R expression and function, improves oxidative stress and inflammatory/anti-inflammatory balance, therefore, lowers blood pressure and recovers renal function in ZDF rats.

**Electronic supplementary material:**

The online version of this article (doi:10.1186/s12933-015-0239-7) contains supplementary material, which is available to authorized users.

## Background

Type 2 diabetes and hypertension are two of the most common diseases and their incidences are increasing dramatically worldwide with concomitant obesity [1]. Obesity promotes insulin resistance, which may further contribute to development of type 2 diabetes and hypertension [2]. However, the mechanisms involved in obesity-related development of hypertension and accompanying complications are not clearly understood. Hypertension in patients and animal models with obesity and insulin resistance is usually associated with increased sodium retention, leading to development of hypertension [3].

Renin-angiotensin-aldosterone system (RAS) plays a critical role in the regulation of renal sodium excretion through a variety of physiological pathways [4, 5]. Angiotensin II (ANG II) is the main effector peptide of RAS, which mediates its antinatriuretic effects via ANG II type 1 (AT_1_) receptors [6], whereas activation of ANG II type 2 (AT_2_) receptors produces natriuresis [6]. Within the kidney, 95 % of the receptors are of AT_1_ subtype, altered functioning of which has been linked to various forms of hypertension [7]. In spontaneously hypertensive rats (SHR), a commonly used animal model of human essential hypertension, and ZDF rats, old Fischer 344 x Brown Norway F1 hybrid rats, the high blood pressure is associated with renal AT_1_ receptor up-regulation [8–10]. However, the mechanisms leading to the up-regulated renal AT_1_R expression and function are not clear.

Although the mechanisms of obesity-related hypertension are complicated, hypertension and obesity, to some extent, are taken as inflammatory diseases. Recent studies suggest that NF-κB is the major transcription factor for AT_1_R gene [11]. Preliminary computer analysis of the AT_1_R 5′-flanking region (GenBank^TM^accession number S66402) has revealed two putative NF-κB binding sites at-365 and-2540 [12]. There are reports showing that NF-κB activation could increase AT_1_R expression [13]. For example, previous studies have shown that ANG II infusion activates NF-κB in the hypothalamic paraventricular nucleus (PVN) and increases hypertensive response, which are associated with the increases of AT_1_R expression in the PVN [14]. Central blockade of NF-κB attenuates blood pressure, and decreases NF- κB activation and AT_1_R expression in the PVN of ANG II-infused rats [14], suggesting an interaction between NF-κB activation and AT_1_R in the cardiovascular regulatory centres. Our previous studies demonstrate that blockade of NF-κB with a NF-κB inhibitor, would decrease AT_1_R expression and decrease augmented AT_1_R-mediated vasoconstriction and sodium retension G-protein–coupled receptor kinase 4 (GRK4) γ variant 142 V, a hypertensive transgenic animal model [15]. Therefore, we wonder whether or not blockade of NF-kB would reduce the AT_1_R expression and reverse the AT_1_R-mediated augmented sodium retention, and then reduce blood pressure in ZDF rats. Our present study would use pyrrolidine dithiocarbamate (PDTC) to treat ZDF rats, observe the effect of PDTC on AT_1_R expression and function in kidney, and investigate the possible underlying mechanisms. PDTC, a NF-κB inhibitor, is believed to exert its inhibitory effects on NF-κB/DNA-binding activities by directly impeding IκBα degradation and IκBα phosphorylation, precluding the dissociation of NF-κB from IκB and subsequent NF-κB translocation from the nucleus [16].

## Materials and methods

### Animals

Male Zucker diabetic fatty (ZDF) and age matched lean Zucker (LZ) rats (Charles River Laboratory, Wilmington, MA) were housed in a temperature-controlled room under a 12/12 h light/dark cycle and had free access to food and water. These experiments were reviewed and approved by The Third Military Medical University Animal Care and Use Committee and conformed to the Guidelines for the Guide for the Care and Use of Laboratory Animals published by the US National Institutes of Health (NIH Pub-lication No. 85–23, revised 1996).

### Experimental protocol

ZDF and LZ rats were randomized into two groups respectively at the age of 12 weeks: control group and group treated with PDTC. Control rats were received treatment with vehicle (tap water) for 4 weeks, while, PDTC group treated with PDTC (150 mg · kg boby wt^−1^ · day^−1^), dissolved in drinking water; the treatment period was lasted for 4 weeks.

Blood pressure for all animals was measured at baseline (12 week-old of age) and then every week until the end of the study by tail-cuff plethysmography (BP-98A; Softron, Tokyo, Japan) method. During experiment, the rats were placed in metabolic cages for 24 h urine collection. Normal tap water for drinking was provided *ad libitum*, daily 24-h water intake and urine volume and sodium excretion were recorded during the study. Before sacrificed, fasting blood glucose was measured using a commercially available glucometer (Roche Diagnostics, Indianapolis, IN). Insulin level was determined by a rat insulin ELISA kit (Millipore, Darmstadt, Germany). Triglycerides were measured by a triglyceride analyzer (Polymer Technology Systems, Cardiochek, IN). Urinary albumin was quantified using the Nephrat kit according to manufacturer’s instructions.

### Surgical procedures for renal function studies

The rats were anesthetized with pentobarbital (50 mg/kg, intraperitoneally; Sigma), and a tracheotomy (PE-240) was performed to facilitate breathing. For measurement of systemic arterial pressure and heart rate, catheters (PE-50) were placed into carotid artery and connected to a pressure transducer (Grass Instrument, Quincy, MA). The left jugular vein was catheterized with PE-50 tubing to infuse normal saline for fluid replacement. A midline abdominal incision was made, the right suprarenal artery was catheterized (PE-10); and the vehicle (saline)/reagents were infused at a rate of 40 μl/h, and both the right and left ureters were catheterized (PE-10) to collect urine. The duration of surgical preparation was about 60 min. To maintain a stable urinary output, 5 % albumin was used to replace blood extracted during each period and normal saline solution equal to 1 % body weight per hour for insensible fluid loss was maintained. Rats were allowed to stabilize for 120 min after surgery prior to 40-min urine collections for clearance measurements.

### Experimental protocol for renal function studies

For determination of effect of candesartan-mediated diuresis and natriuresis, rats were stabilized for 120 min after surgery and followed by five consecutive 40-min collection periods; basal, reagent treatment (3 periods), recovery. During basal, vehicle alone was infused through the right suprarenal artery; during reagent treatment period, candesartan (10 μg/kg/min, i.v.), an AT_1_ receptor antagonist was infused; and during recovery, only vehicle was infused. Blood samples (200 μl; replaced by equal volume of 5 % albumin) were collected at the end of each period. At the end of the experiment, plasma was separated by centrifuging blood samples at 1500 g for 15 min at 4 °C. Sodium and potassium concentrations in urine samples were measured by a flame photometer 480 (Ciba Corning Diagnostics, Norwood, MA). Creatinine levels in the plasma and urine were measured by a creatinine analyzer (Beckman, Fullerton, CA). The glomerular filtration rate (GFR) (milliliters per minute) was calculated from creatinine clearance [17].

### Renal cortex IL-β, IL-10, TNF-α measurements

IL-β, IL-10 and TNF-α in the renal cortex were measured by ELISA using a commercially available kit (Boster, Wuhan, China) according to the manufacturer’s protocol.

### Activity of NADPH oxidase by lucigenin-enhanced chemiluminescence

The NADPH oxidase activity was measured by the lucigenin-enhanced chemiluminescence method [18]. Briefly, NADPH (100 μM, Sigma) and lucigenin (5 μM, Sigma) were added into 1 ml microcentrifugal tubes. Superoxide production was measured every 20 s for 10 min and values were expressed as relative luminescence units per minute per milligram of protein. Using this method, the superoxide anion production also represents NADPH oxidase activity.

### Real-time quantitative RT-PCR (qRT-PCR) analysis

qRT-PCR was used to determine mRNA levels of AT_1_R; oxidative stress markers viz. gp91phox (also known as NOX2), and iNOS in the renal cortex by using specific primers. The primer sequences used for qRT-PCR were listed in the Additional file 1: Table S1. Total RNA was isolated from the kidneys using SV total RNA isolation system from Promega (Madison, WI), and cDNA was synthesized using reverse transcript reagents from Bio-Rad Laboratories (Hercules, CA). The mRNA level was quantified using Bio-Rad iCyCler real-time PCR machine. Gene expression was measured by the ΔΔCT method and was normalized to GAPDH mRNA levels. The data were presented as the fold change of the gene of interest relative to that of control animals.

### Nuclear/cytosolic fractionation

Nuclear and cytosolic proteins were extracted from renal cortex, using NE-PER reagents (Thermo Scientific, Lafayette, CO). Briefly the cortex homogenates were suspended in CER I lysis buffer, CER II buffer was added and further vortexed to ensure complete mixing. The cortex suspension was centrifuged and the supernatant yielded the cytosolic fraction. NER I buffer was added and further vortexed for nuclear membrane lysis. The suspension was centrifuged and the supernatant collected as the nuclear fraction. Total protein in both fractions was determined by BCA assay, and equivalent proteins were loaded.

### Western blot analysis

The expression of AT_1_R, NF-κB p65 and phosphorylation of IKKα/β, IκBα, oxidative stress markers (NOX2, iNOS), and GAPDH in the renal cortex were determined by Western blotting. The renal cortices were homogenized in ice-cold lysis buffer (PBS with 1 % NP40, 1 mmol/L EDTA, 1 mmol/L PMSF, 10 μg /ml leupeptin and 10 μg/ml aprotinin inhibitor). Equal amounts of total extracted proteins (50 μg) were separated on SDS-PAGE and were transferred onto nitrocellulose membranes (Amersham Life Science, Arlington, TX). The blots were subjected to immunoblot analyses with the primary polyclonal antibodies for rabbit anti-AT_1_R, anti-IKKα/β, phospho-IKKα/β, anti-IκBα, phospho-IκBα, NOX2 and iNOS (1:300; Santa Cruz Biotechnology, Santa Cruz, CA), anti-NF-κB p65 (1:400; BD Transduction Laboratory, Minneapolis, MN, USA), anti-Histone and anti-GAPDH (1:500, Santa Cruz Biotechnology). Immunodetection was accomplished by incubating the blots in horseradish peroxidase-conjugated anti-rabbit secondary antibody (1:10,000 dilution). The bands were visualized using enhanced chemiluminescece kit (Amersham, Arlington, TX), and the band intensities were quantified by densitometry using Quantity-One software (Bio-Rad, Hercules, CA), and normalized with GAPDH expression.

### Electrophoretic mobility shift assay (EMSA)

The NF-κB-DNA binding activity in renal cortex was measured by EMSA. EMSA was performed with the Light-shift Chemilunminescent EMSA kit (Prerce Chemical Co, Rockford, IL) according to the manufacturer’s protocol. A synthetic double-stranded oligonucleotides probe (NF-κB:5′-AGTTGAGGGGACTTTCCCAGGC-3′) containing the rat AT_1_R gene promoter with the sequence between nucleotides −350 bp and −363 bp (5′-AAGGGAGTTCCCTA-3′), and NF-κB mutant oligonucleotides (5′-AGTTGAGGGATCTTTCCCAGGC-3′) were labeled with biotin and incubated with the nuclear extracts.

### Statistical analysis

Data are expressed as the mean ± SEM. Statistical significance between experimental groups was determined using the unpaired t test or ANOVA with Newman-Keuls multiple test, as appropriate. Statistical analysis was carried out using a software program (GraphPad Prism version 5; GraphPad Software, San Diego, CA). *P* < 0.05 was considered statistically significant.

## Results

### Physiological parameters

**Fig. 1:**
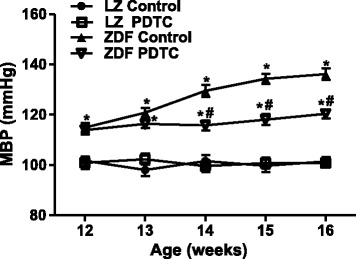
Effect of PDTC on blood pressure in LZ and ZDF rats. Mean blood pressure (MBP ) was recorded in LZ and ZDF rats with different ages (12–16 weeks). The rats were treated with PDTC (150 mg · kg body wt-1 · day-1) or vehicle for 4 weeks. ^*^ *P* <0.05 vs LZ control; ^#^ *P* < 0.05 vs ZDF control (*n* = 8)

In order to determine the metabolic characteristics of the LZ and ZDF rats, we measured their body weight, plasma levels of insulin, glucose, triglyceride and blood pressure. As shown in Table 1, body weight, food intake, plasma levels of insulin, glucose and triglyceride were significantly higher in ZDF than in LZ rats, but heart rate had no difference. PDTC treatment for 4 weeks lowered plasma levels of insulin, glucose and triglyceride in ZDF rats, but not in LZ rats. As expected, the blood pressure was significantly higher in ZDF than LZ rats at baseline. PDTC treatment prevented the increase in blood pressure with age in ZDF rats although the blood pressure remained higher than those observed in lean Zucker rats (Fig. 1).

**Table 1:** Physiological parameters

Parameter	LZ Control	LZ PDTC	ZDF Control	ZDF PDTC
Food intake (g/day)	23.4 ± 1.4	24.0 ± 1.7	37.7 ± 1.6^*^	36.7 ± 1.2^*^
Body weight (g)	290.1 ± 6.5	288.6 ± 4.3	435.3 ± 7.3^*^	431.1 ± 7.5^*^
Blood glucose (mmol/L)	5.5 ± 0.2	5.3 ± 0.3	8.6 ± 0.3^*^	7.0 ± 0.1^*#^
Insulin (nmol/L)	0.63 ± 0.04	0.60 ± 0.04	4.17 ± 0.20^*^	1.67 ± 0.14^*#^
Triglycerides (mg/dl)	61.8 ± 5.0	57.4 ± 4.3	315.9 ± 13.3^*^	132.2 ± 15.5^*#^
Heart rate (bpm)	370.6 ± 5.9	367.8 ± 5.6	363.3 ± 4.2	362.4 ± 4.6

Effect of PDTC on physiological characteristics in LZ and ZDF rats. Data are expressed as mean ± SEM (*n* = 8/group). *P* < 0.05 was considered statistically significant. ^*^ *P* <0.05 vs. LZ control; ^#^ *P* < 0.05 vs. ZDF control

### Effect of PDTC on renal function in ZDF rats

Compared with LZ rats, ZDF rats consumed more water over 24 h urine collection period. PDTC treatment did not affect water intake in both rat strains. As indicator of renal insufficiency, as compared with LZ rats, ZDF rats had increased renal weights, plasma creatinine, and urine albumin excretion, urinary volume output and sodium excretion. After adjusting for body weight, urinary volume output and sodium excretion were indeed lower in ZDF than LZ rats. Moreover, ZDF rats had lower glomerular filtration rate (GFR) than ZL rats. PDTC treatment significantly prevented the increased plasma creatinine and urinary albumin, normalized urinary volume output, urinary sodium excretion and GFR in ZDF rats (Table 2).

**Table 2:** Renal function analysis

Group	LZ Control	LZ PDTC	ZDF Control	ZDF PDTC
Kidney weight (g)	1.02 ± 0.03	1.01 ± 0.02	1.83 ± 0.04^*^	1.80 ± 0.03^*^
Water intake (ml/day)	17.3 ± 0.64	16.9 ± 0.84	22.3 ± 0.69^*^	21.8 ± 0.74^*^
Creatinine (mg/dl)	0.62 ± 0.05	0.63 ± 0.05	1.25 ± 0.13^*^	0.77 ± 0.04^#^
Urinary albumin (mg/24 h)	10.69 ± 0.97	10.36 ± 1.55	60.49 ± 4.93^*^	36.19 ± 3.23^*#^
Urine Volume (ml/day)	7.09 ± 0.48	6.83 ± 0.64	8.28 ± 0.50^*^	10.02 ± 0.90^*#^
Normalized urine volume (ml/day/kg · body wt)	24.74 ± 2.17	24.10 ± 2.44	19.42 ± 1.50^*^	23.95 ± 1.21^#^
UNaV (mmol/day)	224.5 ± 13.3	227.8 ± 20.4	264.4 ± 11.4^*^	298.9 ± 14.5^*#^
Normalized UnaV (μmol/day /kg · body wt)	782.8 ± 62.9	801.8 ± 74.5	596.3 ± 37.8^*^	730.1 ± 58.2^#^
GFR (ml/min)	1.01 ± 0.04	0.96 ± 0.05	0.75 ± 0.03^*^	0.88 ± 0.04^#^

Effect of PDTC on renal function in LZ and ZDF rats. Data are expressed as mean ± SEM (*n* = 5/group). *P* < 0.05 was considered statistically significant. ^*^ *P* <0.05 vs. LZ control; ^#^ *P* < 0.05 vs. ZDF control

### Effect of PDTC on inflammation and oxidative stress in kidney of ZDF rats

**Fig. 2:**
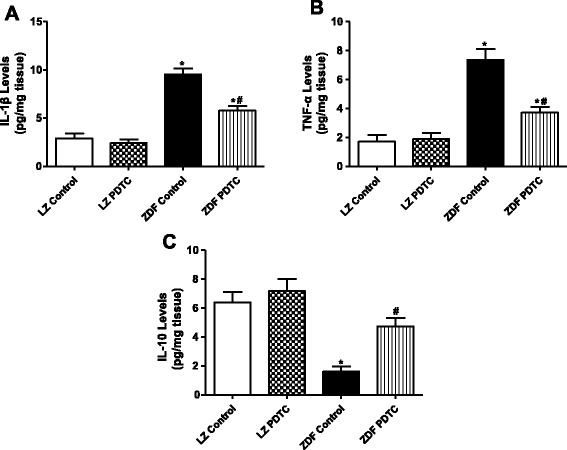
Effect of PDTC on the levels of inflammatory markers in the renal cortex of LZ and ZDF rats. The inflammatory markers included IL-1β (**a**), TNF-α (**b**) and IL-10 (**c**). The rats were treated with PDTC (150 mg · kg body wt-1 · day-1) or vehicle for 4 weeks. ^*^ *P* <0.05 vs LZ control; ^#^ *P* < 0.05 vs ZDF control (*n* = 6)

Given role of inflammation and oxidative stress in the development of type-2 diabetes and hypertension, we checked inflammation and oxidative stress in those rats. It showed that the increase in blood pressure was accompanied by an increased production of local proinflammation and a reduction of anti-inflammatory makers in ZDF rats than LZ rats, i.e., ZDF rats had an increased abundance of IL-1β, TNF-α, and a decreased level of IL-10 (Fig. 2a and c).

**Fig. 3:**
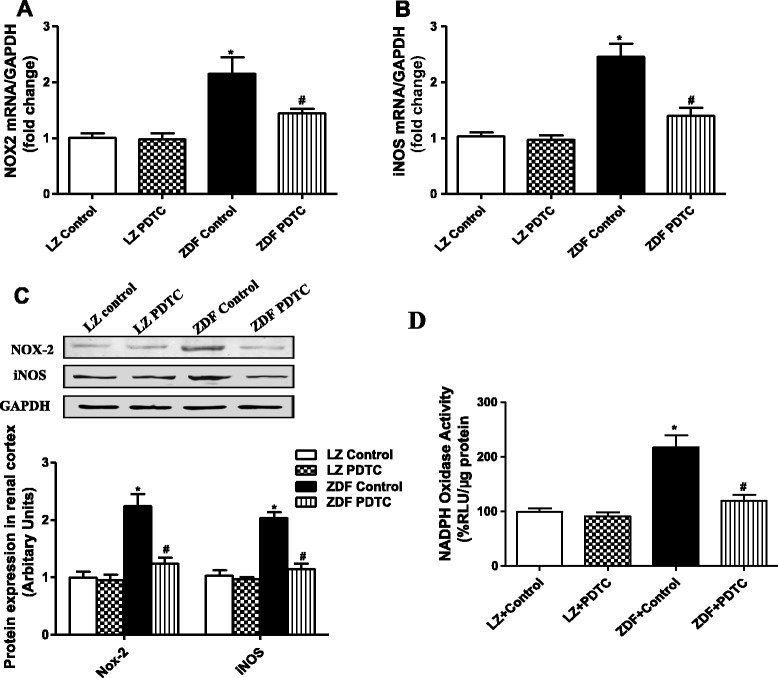
Effects of PDTC on Nox-2 and iNOS expression in the renal cortex of LZ and ZDF rats. mRNA level of Nox-2 (**a**) and iNOS (**b**) in the renal cortex was measured using qRT-PCR and normalized to GAPDH expression. **c** Protein expression of Nox-2 and iNOS in the renal cortex of LZ and ZDF rats was measured by western blot, and data were normalized using GAPDH expression. **d** The activity of NADPH oxidase in renal cortical homogenates was measured by using lucigenin-enhanced chemiluminescence and expressed as percentage of relative luminescence units (RLU)/μg protein. *P <0.05 vs LZ control; ^#^ *P* < 0.05 vs ZDF control (*n* = 5)

Consistent with inflammatory change, oxidative stress was also increased in ZDF rats. NOX2, a subunit of NAD(P)H oxidase as oxidative stress marker, and inducible nitric oxide synthase (iNOS) were significantly higher in the renal cortices of ZDF rats as compared with LZ rats (Fig. 3a and c). In order to confirm that the increase on iNOS and Nox-2 expression correlates with an increase in the activity, the activity of NADPH oxidase in renal cortices was measured by using lucigenin-enhanced chemiluminescence (Fig. 3d). The NADPH oxidase activity was increased by 221 ± 43 % in ZDF compared to LZ rats. PDTC treatment normalized the above-mentioned abnormal inflammation and oxidative stress in ZDF rats.

### Effect of PDTC on AT_1_ receptor expression and function in ZDF rats

**Fig. 4:**
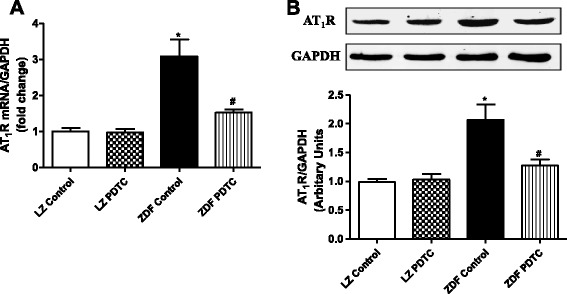
Effect of PDTC on the expressions of AT_1_R mRNA and protein in renal cortex of LZ and ZDF rats. mRNA expression in the renal cortex was measured using qRT-PCR and normalized to GAPDH expression (**a**). Protein expression was measured by western blot using specific antibodies against AT_1_R and data were normalized using GAPDH expression (**b**). ^*^ *P* <0.05 vs LZ control; ^#^ *P* < 0.05 vs ZDF control (*n* = 5)

Due to the important role of AT_1_R on renal function, we determined its expression in kidney, it resulted that AT_1_R protein and mRNA expressions were higher in renal cortex from ZDF rats than LZ rats. PDTC treatment for 4 weeks significantly decreased AT_1_R expression in kidney from ZDF rats, not from LZ rats (Fig. 4a and b).

**Fig. 5:**
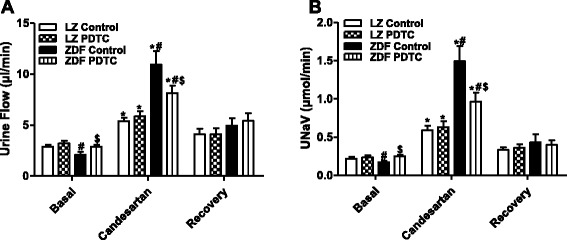
Effect of PDTC on AT_1_ receptor function in LZ and ZDF rats. LZ or ZDF rats were treated with PDTC (150 mg · kg body wt-1 · day-1) or vehicle for 4 weeks. Adjusting for kidney weight, urine flow (**a**) and urinary sodium excretion (UNaV) (**b**) were recorded during the vehicle or candesartan (10 μg/kg body wt per min) infusion via the right supraenal artery of anesthetized rats. Values of three durg (drug 1, drug 2, and drug 3) collections were averaged and were shown. Results are shown as mean ± SEM (*n* = 6/group). ^*^ *P* < 0.05 vs respective basal; ^#^ *P* < 0.05 vs lean control within the same treatment; ^$^ *P* < 0.05 vs obese control within the same treatment

The increased AT_1_R was pathophysiological significance. AT_1_R antagonist candesartan-mediated natriuretic and diuretic effects were significantly higher in ZDF rats than in LZ rats. Consistent with effect of PDTC on AT_1_R expression, PDTC treatment normalized candesartan-mediated natriuresis and diuresis in ZDF rats (Fig. 5a and b). Heart rate and blood pressure were not significantly different in all four groups of rats during candesartan infusion (Additional file 2: Figure S1). To determine if there was any systemic effect of the drugs selectively infused into the right suprarenal artery, urine flow and sodium excretion from the left kidney were also measured. We found that the renal function, including urine flow and sodium excretion, in the left unperfused kidney was not altered by any of the drug treatments (data not shown).

### PDTC inhibited the activation of NF-κB signaling and reduced NF-κB–binding activity in ZDF rats

**Fig. 6:**
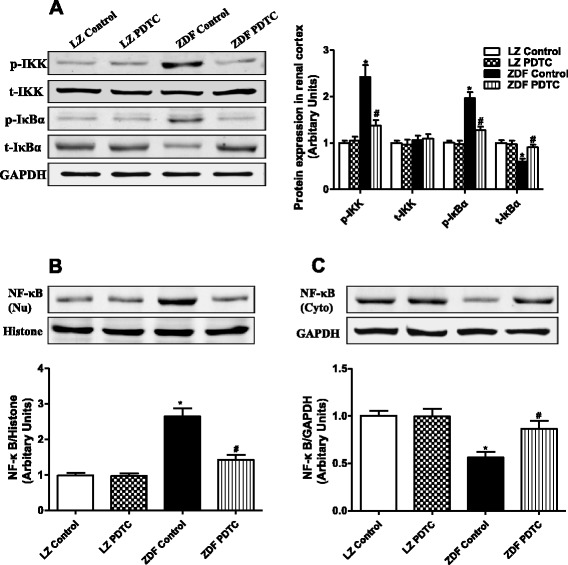
Effect of PDTC on NF-κB signaling in the renal cortex of LZ and ZDF rats. LZ or ZDF rats were treated with PDTC (150 mg · kg body wt-1 · day-1) or vehicle for 4 weeks. **a**: Phosphorylation and expression of IKK and IκBα were determined by Western blot, and data were normalized using GAPDH expression. Nuclear and cytosol protein were prepared from the renal cortex and the expression of NF-κB in nuclear (Nu) (**b**) and cytosol (Cyto) (**c**) fractions were determined by Western blot. ^*^ *P* <0.05 vs LZ control; ^#^ *P* < 0.05 vs ZDF control (*n* = 5)

**Fig 7:**
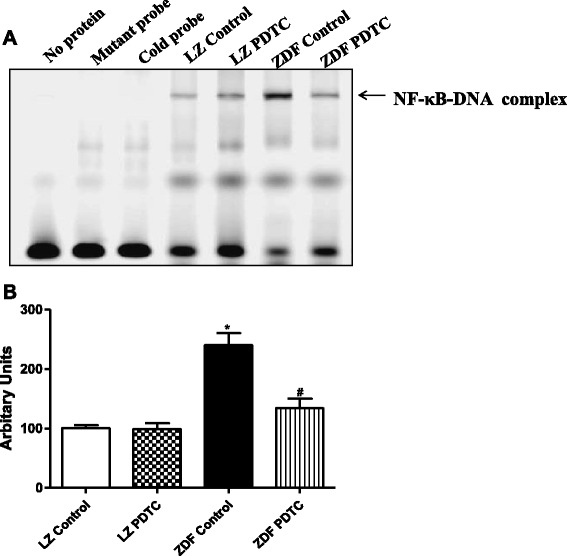
Effect of PDTC on NF-κB-DNA binding activities in the renal cortex of LZ and ZDF rats. Binding activity of NF-κB was examined in nuclear proteins from the renal cortex of LZ and ZDF rats by Electrophoretic Mobility Shift Assay (EMSA). **a**: DNA-binding ability of NF-κB to the promoters of AT_1_R gene. No nuclear extracts (negative controls) (lane 1), mutant probe (lane 2) and cold probe (lane 3). **b**: Densitometric analysis of NF-κB-DNA binding activities in the renal cortex from each group of rats. ^*^ *P* <0.05 vs LZ control; ^#^ *P* < 0.05 vs ZDF control (*n* = 5)

It is known that NF-κB is the vital signaling of oxidative stress and inflammation on AT_1_R expression, we checked NF-κB related pathway, it showed that there was a marked increase in phosphorylation of IKK and IκBα in ZDF rats, which was accompanied by decreased protein expression of IκBα (Fig. 6a). Nuclear translocation of NF-κB was also observed in the kidney of ZDF rats. This was reflected by increased expression of NF-κB p65 subunits, in the nuclear fraction, and decreased expression of NF-κB p65 subunits in the cytosolic fraction (Fig. 6b and c). The binding activity of NF-κB binding to the AT_1_R promoter was significantly higher in renal cortices of ZDF than in LZ rats by EMSA analysis (Fig. 7). As an inhibitor of NF-κB, PDTC inhibited phosphorylation of IKK and IκBα, increased IκBα expression, blocked the translocation of NF-κB from cytosolic to nuclear fraction, therefore, decreased the binding of NF-κB with AT_1_R promoter, and decreased AT_1_R expression in kidney from ZDF rats (Fig. 6 and 7).

## Discussion

In this study, we examined the effects of chronic NF-κB blockade with PDTC on kidney cortical inflammatory cytokines production, oxidative stress and renal AT_1_ receptor expression and function in ZDF rats. The salient findings of the present study are 1) the upregulation of NF-κB contributed to AT_1_ receptor dysfunction and increased inflammation and oxidative stress in ZDF rats. 2) NF-κB blockade improved the balance between pro- and anti-inflammatory cytokine by attenuating proinflammatory cytokine (TNF-α, IL-1β) and upregulating anti-inflammatory IL-10, and attenuated oxidative stress (Nox-2, iNOS) in the renal cortex of ZDF rats. 3) NF-κB blockade attenuated blood pressure partially by reducing AT_1_R expression and normalizing renal AT_1_ receptor function in ZDF rats. These data suggest that NF-κB plays an important role in renal AT_1_ receptor function in ZDF rats by increasing the binding of NF-κB with AT_1_R promoter, and NF-κB blockade reduces AT_1_R expression and function in kidney, subsequently improves sodium excretion, lowers blood pressure in ZDF rats.

### The contribution of metabolic factor to hypertension: role of NF-κB

A variety of different factors probably contribute to the pathogenesis of hypertension in ZDF rats. The ZDF rats, a model of metabolic syndrome, which is typified by hyperglycemia, hyperinsulinemia, hyperlipidemia, and insulin resistance [19]. Hyperglycemia is a key initiator of the cardiovascular complications associated with diabetes mellitus. Hyperglycemia leads to an increase in oxidative stress, by exacerbating glucose oxidation and inducing activation of renal RAS components [20]. Insulin induces cardiac and renal hypertrophy and remodeling during insulin resistance and hyperinsulinemia in type 2 diabetes by NF-κB activation, which may further contribute to development of hypertension [21]. Hyperlipidemia-induced intracellular generation of ROS can act as signal transduction molecules to activate various signaling pathways, which ultimately lead to inflammation [22]. Increased lipidemia has been consistently associated with renal damage, and the NF-κB-blocking properties of PDTC led to significant decreases in plasma lipids [23]. In addition, recent study has demonstrated that vaspin and adiponectin are significantly decreased in metabolic syndrome, which may lead to cytokine-induced NF-κB activation, increasing inflammatory response and oxidative stress [24, 25]. In accordance with the observations of previous studies, our present results showing PDTC treatment improved insulin sensitivity, reduced plasma insulin and normalized blood glucose levels, decreased blood pressure in ZDF rats. Therefore, we speculate the possibility that the decrease in circulating insulin, glucose and plasma lipids with PDTC treatment can further decrease the inflammatory status and oxidative stress, and could be also responsible for reducing blood pressure in ZDF rats.

### Role of NF-κB activation in altering AT_1_R expression and function

NF-κB plays an important role in the pathogenesis of cardiovascular diseases, including hypertension. However, the mechanism by which NF-κB in the kidney contributes to the progression of hypertension is not known. Hypertension is characterized by impaired sodium handling in kidney [26]. AT_1_R plays a vital role in this process [27]. The upregulation of AT_1_R could promote sodium retention and lead to development of hypertension [7]. It has been reported that there is a marked increase in the AT_1_R expression and function in ZDF rats compared with LZ rats [9, 28, 29]. We hypothesize that NF-κB activation promotes the exaggerated expression and function of renal AT_1_ receptor and contributes to hypertensive response in ZDF rats. Recent studies suggest that NF-κB, a redox-sensitive transcription factor, upregulates AT_1_ receptor involving two binding sites within the 5′-flanking region of AT_1_ receptor gene. Also, NF-κB is necessary for cytokine-induced upregulation of both AT_1_ receptor mRNA and protein expression [11, 30]. NF-κB blockade have attenuated expression of AT_1_R protein and mRNA in the PVN of ANG II-infused rats, and normalized Ang II-induced vasoconstriction in SHR, suggesting an interaction between RAS and NF-κB in the cardiovascular regulatory centres [14]. This is in agreement with earlier studies [29, 31, 32], we also found increased nuclear levels of NF-κB and AT_1_R expression in renal cortices of ZDF rats. Since NF-κB has been activated, we wanted to determine whether blocking NF-κB attenuated the renal AT_1_R function and expression, lowed blood pressure in ZDF rats. Our present results showed that treatment of ZDF rats with PDTC inhibited NF-κB activation, reduced expression and function of AT_1_R and blood pressure in the kidney of these rats, suggesting that NF-κB activation increases AT_1_R expression in the kidney and contributes to hypertension in ZDF rats. Recent study has demonstrated that the activation of glucagon-like peptide-1 receptor can inhibit vascular smooth muscle cells calcification through NF-κB/RANKL signaling [33] and chronic stimulation of AT_4_ and inhibition of AT_2_ receptors reverse diabetes-induced endothelial dysfunction [34], which could also play a vital role in renal sodium handling.

### Association of NF-κB with inflammation and oxidative stress: role of AT_1_R

A growing body of evidence indicates that hypertension is an inflammatory state wherein proinflammatory cytokines, such as tumour necrosis factor-alpha (TNF-α) and interleukin-6 (IL-6), contribute to the hypertensive effect [35]. The NF-κB complex is one of the most important proinflammatory intracellular signaling systems. Activation of NF-κB complex has been linked to an increase in the synthesis of AT_1_R and proinflammatory cytokines [36]. Accordingly, inhibition of the NF-κB system or AT_1_R abolishes the associated inflammatory response [37, 38]. In addition to regulating proinflammatory cytokine synthesis, NF-κB also contributes to NAD(P)H oxidase-dependent oxidative stress [39]. Previous study have demonstrated that activated NF-κB in the kidney induces to NAD(P)H oxidase-dependent oxidative stress and NAD(P)H-dependent superoxide contributed to the mechanism of hypertension by promoting sodium retention [40–42]. Other recent studies indicate that ROS production is increased in humans with hypertension and several hypertensive animal models [43, 44], and oxidative stress upregulates vascular and renal AT_1_R via mechanisms involving NF-κB and chronic AT_1_R blockade significantly reduces an increase of ROS production [45, 46], suggesting an interaction between AT_1_R, NF-κB, and oxidative stress in the cardiovascular regulatory centres. In this study, we found that chronic NF-κB blockade with PDTC might have reduced AT_1_R expression and attenuated the increase in oxidative stress by partially inhibiting the positive feedback between ROS and NF-κB in the kidney of ZDF rats. This suggests that the interaction between oxidative stress and NF-κB plays a critical role in AT_1_R expression and functions of ZDF rats. In our current study, we found that PDTC only prevented further increases in blood pressure with age, but did not reduce the blood pressure to the same as LZ rats, suggesting that although the activation of NF-κB complex is an important, but not the only factor involved in the pathogenesis of hypertension.

## Conclusion

In summary, we show causative role of NF-κB activation in the development of high blood pressure in ZDF rats. The mechanism for this increase in blood pressure may involve NF-κB-mediated alterations in both renal AT_1_ receptor functions and subsequent tubular sodium handling in ZDF rats. Chronic administration of PDTC to ZDF rats restored AT_1_ receptor function and expression, and reduced renal inflammation and oxidative stress, suggesting that inhibition of NF-κB activation may be effective adjuncts to the current treatment of hypertension, although studies of safety and toxicity are required before such drugs can be considered for clinical use.

## Additional files

Additional file 1: Table S1. List of primers used for quantitative real time RT-PCR. Additional file 2: Figure S1. Effect of PDTC on AT_1_ receptor function in LZ and ZDF rats. MBP, mean blood pressure (A) and HR, heart rate (B) were recorded in those rats treated with candesartan (10 μg/kg body wt per min). Values of three durg (durg 1, durg 2, and durg 3) collections were averaged and are shown. ^*^ *P* < 0.05 vs. LZ control rats in the same period, ^#^ *P* < 0.05 vs. ZDF control in the same period (*n* = 6).

## Abbreviations

NF-κB: Nuclear factor-kappa B
AT_1_R: `Angiotensin II type 1 receptor
ZDF: Zucker diabetic fatty
LZ: Lean Zucker
PDTC: Pyrrolidine dithiocarbamate
RAS: Renin-angiotensin-aldosterone system
ANG II: Angiotensin II
PVN: Paraventricular nucleus
GRK4: G-protein–coupled receptor kinase 4
EMSA: Electrophoretic mobility shift assay
TNF-α: Necrosis factor-alpha
IL-6: Interleukin-6
