# Supplementary material for: Chronic NF-κB blockade improves renal angiotensin II type 1 receptor functions and reduces blood pressure in Zucker diabetic rats
Source: Cardiovasc Diabetol. 2015 Jun 10;14:76. doi: 10.1186/s12933-015-0239-7 (PMC4465496; doi:10.1186/s12933-015-0239-7)
Supplement: Additional file 1: Table S1. — List of primers used for quantitative real time RT-PCR. [file 12933_2015_239_MOESM1_ESM.doc]

**Additional file 1**

**Table S1.** List of primers used for quantitative real time RT-PCR.

| Gene | Sence | Antisence |
| --- | --- | --- |
| NOX2 | CGGAATCTCCTCTCCTTCCT | GCATTCACACACCACTCCAC |
| iNOS | **CCTTGTTCAGCTACGCCTTC** | **GGTATGCCCGAGTTCTTTCA** |
| AT_1_R | **CAAAAGGAGATGGGAGGTCA** | **TGACAAGCAGTTTGGCTTTG** |
| GAPDH | AGACAGCCGCATCTTCTTGT | CTTGCCGTGGGTAGAGTCAT |

NOX2, NADPH oxidase 2; iNOS, inducible nitric oxide synthase; AT_1_R, angiotensin II type 1 receptor
