# Supplementary figures and images for: Chronic NF-κB blockade improves renal angiotensin II type 1 receptor functions and reduces blood pressure in Zucker diabetic rats
Source: Cardiovasc Diabetol. 2015 Jun 10;14:76. doi: 10.1186/s12933-015-0239-7 (PMC4465496; doi:10.1186/s12933-015-0239-7)

## Slide 1
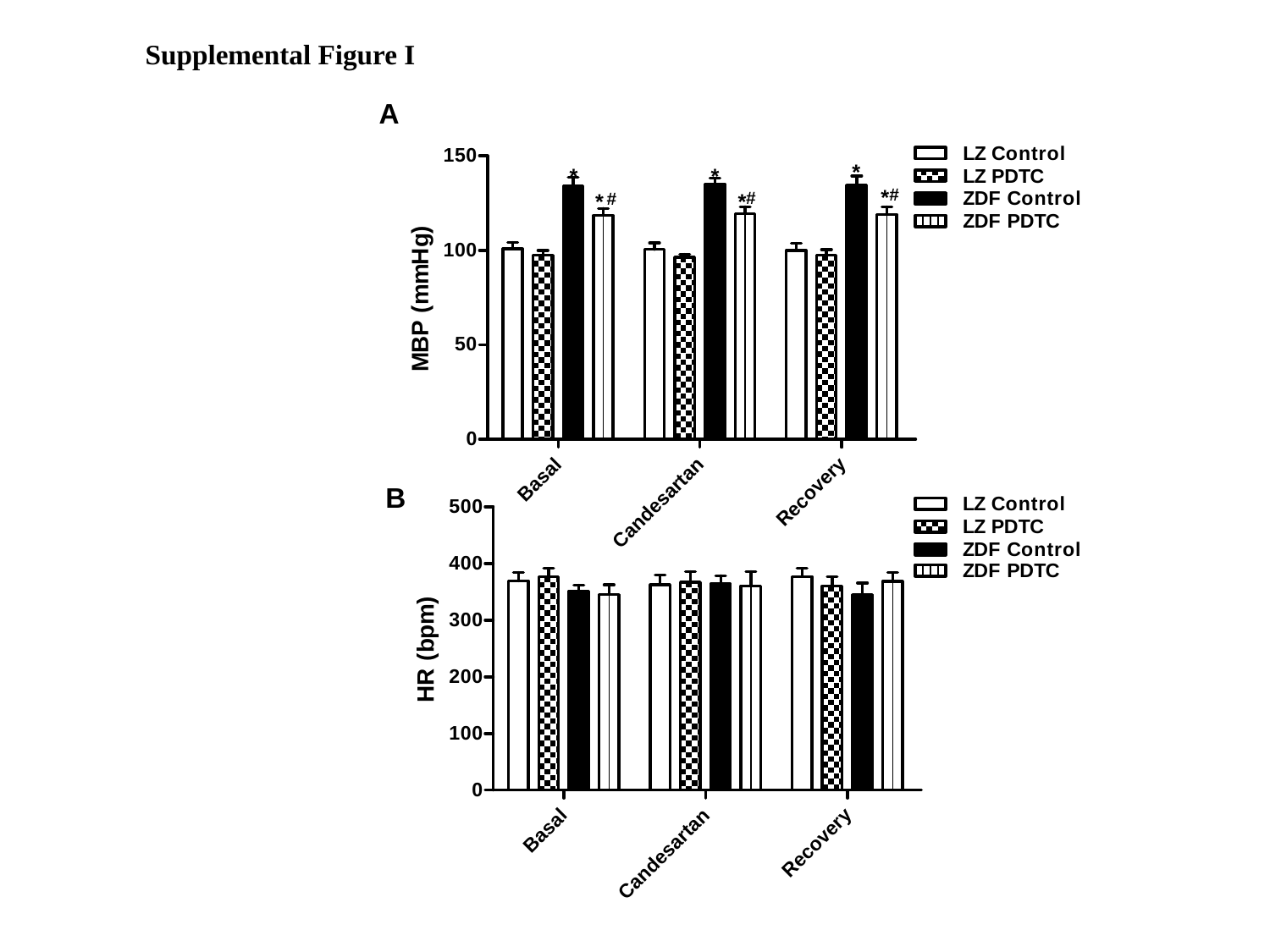

Supplemental Figure I
A
B

Supplement: Additional file 2: Figure S1. — Effect of PDTC on AT1 receptor function in LZ and ZDF rats. MBP, mean blood pressure (A) and HR, heart rate (B) were recorded in those rats treated with candesartan (10 μg/kg body wt per min). Values of three durg (durg 1, durg 2, and durg 3) collections were averaged and are shown. * P < 0.05 vs. LZ control rats in the same period, # P < 0.05 vs. ZDF control in the same period (n = 6). [file 12933_2015_239_MOESM2_ESM.pptx]
